# Use of video technology for teaching in intensive care

**DOI:** 10.1186/2197-425X-3-S1-A869

**Published:** 2015-10-01

**Authors:** I Carroll, N Thomas, J Moore

**Affiliations:** Kings College Hospital, UK, London, United Kingdom; North Manchester General Hospital, Manchester, United Kingdom; Central Manchester Foundation NHS Trust, Manchester, United Kingdom

## Introduction

Intensive care units in the UK are staffed by junior doctors with a range of backgrounds (anaesthesia, emergency and general medicine), often on rotations. Training is provided for practical procedures with an assessment of performance prior to independent practice.

There are national guidelines in place for the insertion and use of central venous catheters (CVCs) [1, 2] however complications persist. The national rate for CVC infection rate is quoted at 1.81 CVC-BSI /1000 CVC-pt days [3]. The rate of inadvertent arterial puncture is quoted at 1/1800 [4].

Use of video in medical education is increasingly common [5] and has been shown to be superior to traditional four-step technique [6].

## Objectives

In response to incidents at Central Manchester Foundation Trust, we produced a series of educational videos to improve our CVC complication rate. Whilst other videos are available, they are of varying quality and we believed it beneficial to demonstrate locally accepted technique, using locally available equipment.

## Methods

Two videos were produced (with patient consent and as part of standard clinical care)

1. Preparation for inserting CVCs
2. Inserting internal jugular line with ultrasound

In view of the rarity of complications and our wish to judge effectiveness of the videos we surveyed doctors starting on intensive care on their perceived improvement in all areas of CVC insertion.

## Results

13 doctors were surveyed before and after watching the videos, 8 doctors were senior level (ST3+) and had inserted more than 50 CVCs, none reported any perceived improvement. 5 doctor were junior level (ST2 or below) and had limited experience inserting CVCs. 80% of junior doctors (n = 4) reported improved perceived knowledge in inserting CVCs, particularly in required monitoring and post-procedure care.

## Conclusion

The use of medical education videos has been proven and we believe we have made a high quality video which will improve patient safety and the training of junior doctors. The video will be available to view in the eposter.

## Grant Acknowledgement

Funding for recording the videos was received by TeleflexUK. No individual involved in producing the videos received funding from CMFT.
